# Increased hepatic CD36 expression with age is associated with enhanced susceptibility to nonalcoholic fatty liver disease

**DOI:** 10.18632/aging.100652

**Published:** 2014-04-09

**Authors:** Fareeba Sheedfar, Miranda MY Sung, Marcela Aparicio-Vergara, Niels J Kloosterhuis, Maria Eugenia Miquilena-Colina, Javier Vargas-Castrillón, Maria Febbraio, René L Jacobs, Alain de Bruin, Manlio Vinciguerra, Carmelo García-Monzón, Marten H Hofker, Jason RB Dyck, Debby PY Koonen

**Affiliations:** ^1^University of Groningen, University Medical Center Groningen, Molecular Genetics, Groningen, The Netherlands; ^2^Cardiovascular Research Centre, Department of Pediatrics, University of Alberta, Edmonton, AB, Canada; ^3^Liver Research Unit, University Hospital Santa Cristina, Instituto de Investigación Sanitaria Princesa, CIBERehd, Madrid, Spain; ^4^Department of Dentistry, University of Alberta, Edmonton, AB, Canada; ^5^Agricultural, Department of Food and Nutritional Science, University of Alberta, Edmonton, AB, Canada; ^6^Dutch Molecular Pathology Center, Department of Pathobiology, Faculty of Veterinary Medicine, Utrecht University, Utrecht, The Netherlands; ^7^IRCCS Casa Sollievo della Sofferenza Hospital, San Giovanni Rotondo, Italy; ^8^UCL - Institute of Liver and Digestive Health, Royal Free Hospital, UK

**Keywords:** CD36, aging, liver, steatosis, inflammation, obesity, NAFLD, NASH

## Abstract

CD36 has been associated with obesity and diabetes in human liver diseases, however, its role in age-associated nonalcoholic fatty liver disease (NAFLD) is unknown. Therefore, liver biopsies were collected from individuals with histologically normal livers (n=30), and from patients diagnosed with simple steatosis (NAS; n=26). Patients were divided into two groups according to age and liver biopsy samples were immunostained for CD36. NAFLD parameters were examined in young (12-week) and middle-aged (52-week) C57BL/6J mice, some fed with chow-diet and some fed with low-fat (LFD; 10% kcal fat) or high-fat diet (HFD; 60% kcal fat) for 12-weeks. CD36 expression was positively associated with age in individuals with normal livers but not in NAS patients. However, CD36 was predominantly located at the plasma membrane of hepatocytes in aged NAS patients as compared to young. In chow-fed mice, aging, despite an increase in hepatic CD36 expression, was not associated with the development of NAFLD. However, middle-aged mice did exhibit the development of HFD-induced NAFLD, mediated by an increase of CD36 on the membrane. Enhanced CD36-mediated hepatic fat uptake may contribute to an accelerated progression of NAFLD in mice and humans. Therapies to prevent the increase in CD36 expression and/or CD36 from anchoring at the membrane may prevent the development of NAFLD.

## INTRODUCTION

The world is experiencing a dramatic increase in numbers of elderly people, rising to an estimated 22% of the world population aged 60 years and older by 2050 [1]. Although population aging is an indicator of improving global health, it has also become one of the century's main health problems. Aging is the leading risk factor for all chronic diseases, accounting for 60% of all deaths worldwide [2]. Thus, to understand how aging increases susceptibility to disease is vital in order to sustain a healthy aging society.

Nonalcoholic fatty liver disease (NAFLD) is, worldwide, the most common liver disease, affecting one-third of the overall population [3]. NAFLD includes a wide spectrum of liver pathologies progressing from benign hepatic steatosis, to nonalcoholic-steatohepatitis (NASH), severe cirrhosis and fibrosis [4]. Growing evidence points towards an increased prevalence of NAFLD in older humans [5]. Indeed, aging is associated with a physiological increase in lipid accumulation in non-adipose tissues, including the liver [6]. Moreover, aging is associated with increased mortality in elderly subjects with NAFLD by significantly enhancing the progression to NASH and fibrosis [7,8]. Although fat may accumulate in the liver as a result of multiple abnormalities of hepatic lipid accumulation [9], few studies have examined the effect of aging on hepatic lipid metabolism [10–14]. As not all study outcomes share a common view on the impact of aging on NAFLD development [14], the cellular mechanisms underlying this age-related fat overload in the liver remain ill-defined.

CD36 facilitates the uptake of long-chain fatty acids (LCFA) across the cell membrane and high levels of expression can contribute to lipid accumulation in heart and skeletal muscle [15]. Enhanced efficiency of LCFA uptake is directly related to increased recruitment of CD36 from intracellular storage sites into the plasma membrane. Increased CD36 expression in human and rodent models is commonly related to insulin resistance and type 2 diabetes, including high-fat (HF) feeding and obesity [16–18] and is also observed in the physiological aging process [19–21]. In the liver, CD36 was long believed to play a very minor role in LCFA uptake [15]. Diets rich in fatty acids have, however, been shown to increase protein expression of CD36 in the liver [18]. Moreover, as we have previously observed, LCFA uptake and lipid deposition in the liver are directly related to CD36 expression, suggesting a causative role for increased CD36 expression in the pathogenesis of type 2 diabetes [18]. Similar findings were observed by several other rodent studies [22,23] and have recently been confirmed in liver biopsies of patients with NAFLD, highlighting the clinical relevance of CD36 in human liver diseases associated with obesity and type 2 diabetes [24]. Its role in age-associated NAFLD is, however, not yet known.

We have, therefore, sought to investigate whether increased CD36 expression underlies the increased susceptibility to the development of NAFLD with age. Our data show that aging is associated with a dramatic increase in CD36 expression in human NAFLD as well as in aged mice on a normal diet. Our data also show that aging, in combination with HF-feeding, triggers the presence of CD36 at the cell surface of hepatocytes, which may contribute to enhanced fat uptake in NAFLD and drive the progression of simple steatosis towards NASH. Therefore, our data suggest that therapies to prevent the increase in CD36 expression and CD36 from anchoring at the membrane may prevent the development of NAFLD.

## RESULTS

### Hepatic CD36 expression profile in humans

**Figure 1:**
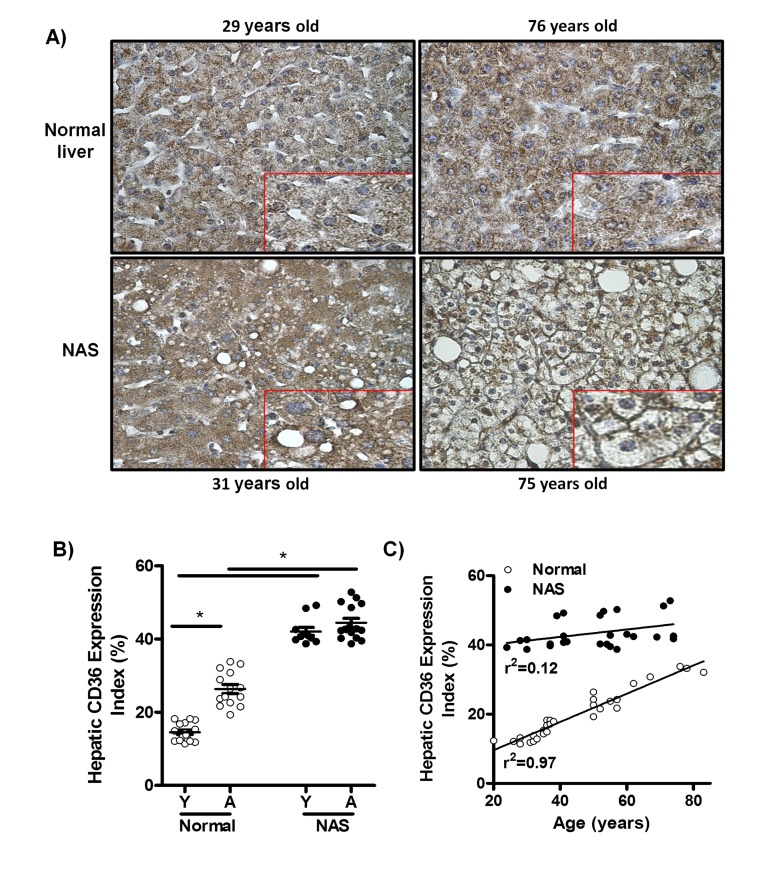
CD36 expression is positively associated with age in liver biopsies from patients with normal livers, but not in liver biopsies from patients with non-alcoholic steatosis (NAS) (**A**) CD36 immunostaining of liver biopsy tissue from: young normal liver, 29 years old (Left upper panel), old normal liver, 76 years old (Right upper panel), young non-alcoholic steatosis (NAS), 31 years old (Left lower panel), old NAS, 75 years old (Right lower panel). Original magnification for all microphotographs: 400x. Each microphotograph includes a 2x zoom detail. (**B**) Hepatic CD36 expression index in liver biopsies from patients with normal livers and NAS. (**C**) Correlation between age and hepatic CD36 expression index in normal livers and NAS.

To determine whether CD36 expression in the human liver is increased by the physiological process of aging, immunohistochemical staining for CD36 was performed in liver biopsy samples from young (20-38 years) and aged individuals (50-83 years) with histologically normal livers, and liver biopsy samples from young (20-42 years) and aged individuals (52-74 years) diagnosed with non-alcoholic steatosis (NAS) (Fig. 1A). We observed a significant increase in the percentage of liver tissue expressing CD36 in aged versus young normal liver sections, suggesting an association between aging and increased CD36 expression in the healthy human liver (Fig. 1B). Furthermore, this increase in CD36 expression in the healthy human liver was directly correlated with age, as shown by linear regression analysis of all normal human liver samples (Fig. 1C; r2=0.97). Aged subjects with normal livers had significant increases in glucose levels, waist perimeters, aspartate aminotransferase and alkaline phosphatase when compared to young subjects (Table 1, p<0.05). However, no differences were observed in body mass index, hip perimeter, plasma lipids, insulin, alanine aminotransferase and gamma-glutamyltransferase between young and aged subjects with normal livers (Table 1). When comparing subjects having normal livers with NAS patients, we also observed in the latter a significant increase in the hepatic CD36 expression index in both young and old (Fig. 1B). However, there was in NAS patients no age-based difference in the CD36 expression (Fig. 1B), suggesting that absolute CD36 protein expression levels in steatotic livers do not correlate with age. Indeed, linear regression analysis confirmed the absence of an age effect on CD36 expression in NAS livers (Fig. 1C; r2=0.12). Further-more, CD36 was largely confined to the cytoplasm of most hepatocytes in both young and aged healthy human liver biopsies (Fig. 1A, upper panels, inserts). Consistent with this, CD36 was also found mainly in the cytoplasm of hepatocytes of young NAS patients (Fig. 1A, left lower panel, insert). This in contrast to aged steatotic livers, where CD36 was located predominantly at the plasma membrane of hepatocytes (Fig. 1A, right lower panel, insert), suggesting that aging may significantly enhance CD36 translocation in steatotic livers. From a clinical point of view, characteristics of young and aged NAS patients were not significantly different (Table 1).

**Table 1:** Characteristics of patients with normal liver and non-alcoholic steatosis (NAS)

	Normal liver		NAS	
	Young (n=16)	Aged (n=14)	Young (n=11)	Aged (n=15)
Age (years)	29 (20 - 38)	67 (50 - 83)*	33 (24 - 42)	63 (52 - 74)*
Female gender (%)	16 (100%)	8 (57%)	7 (64%)	7 (47%)
Body mass index (kg/m^2^)	25.1 ± 4.3	28.2 ± 5.1	28.3 ± 3.6	30.6 ± 4.1
Waist Perimeter (cm)	86.9 ± 8.3	98.2 ± 10.3*	99.0 ± 9.5**	105.3 ± 9.7
Hip Perimeter (cm)	102.3 ± 7.7	104.4 ± 12.1	106.4 ± 8.8	109.7 ± 9.2
Glucose (mg/dl)	85.8 ± 7.7	94.8 ± 10.4*	88.5 ± 7.2	94.1 ± 8.5
Insulin (mU/ml)	6.4 ± 4.1	7.1 ± 2.4	9.5 ± 4.7	9.2 ± 4.2
HOMA-IR	0.8 ± 0.5	0.9 ± 0.3	1.2 ± 0.6	1.2 ± 0.6
Triglycerides (mg/dl)	84.6 ± 32.8	109.6 ± 52.2	143.6 ± 80.9**	123.1 ± 48.0
Total Cholesterol (mg/dl)	180.9 ± 26.9	178.7 ± 41.3	207.3 ± 67	200.6 ± 21.4
HDL-cholesterol (mg/dl)	49.4 ± 8.5	48.5 ± 12.3	48.7 ± 12.3	47.0 ± 10.4
ALT (IU/L)	14.7± 5.5	19.9 ± 9.1	24.5 ± 11.3**	24.4 ± 10.4
AST (IU/L)	15.3 ± 3.4	18.8 ± 4.4*	19.7 ± 8.4	21.1 ± 4.3
-GT (IU/L)	22.7 ± 16.3	33.2 ± 24.7	61.5 ± 79.6	44.9 ± 56.9
ALP (IU/L)	59.8 ± 18.2	77.7 ± 23.9*	60.7 ± 21.8	79.7 ± 20.5*
Grade of steatosis				
Grade 0	16 (100%)	14 (100%)		
Grade 1			9 (82%)	10 (62%)
Grade 2			2 (18%)	5 (38%)

Data are shown as mean ± SD, median (range), or n (%).

* p<0.05 aged vs young

** p<0.05 normal liver young vs NAS young

NAS, non-alcoholic steatosis; HDL, high-density lipoprotein; HOMA-IR, homeostatic model assessment-insulin resistance; ALT, alanine aminotransferase; AST, aspartate aminotransferase; -GT, gamma-glutamyltransferase; ALP, alkaline phosphatase

### Aging does not induce hepatic steatosis or inflammation in chow-fed mice

**Figure 2:**
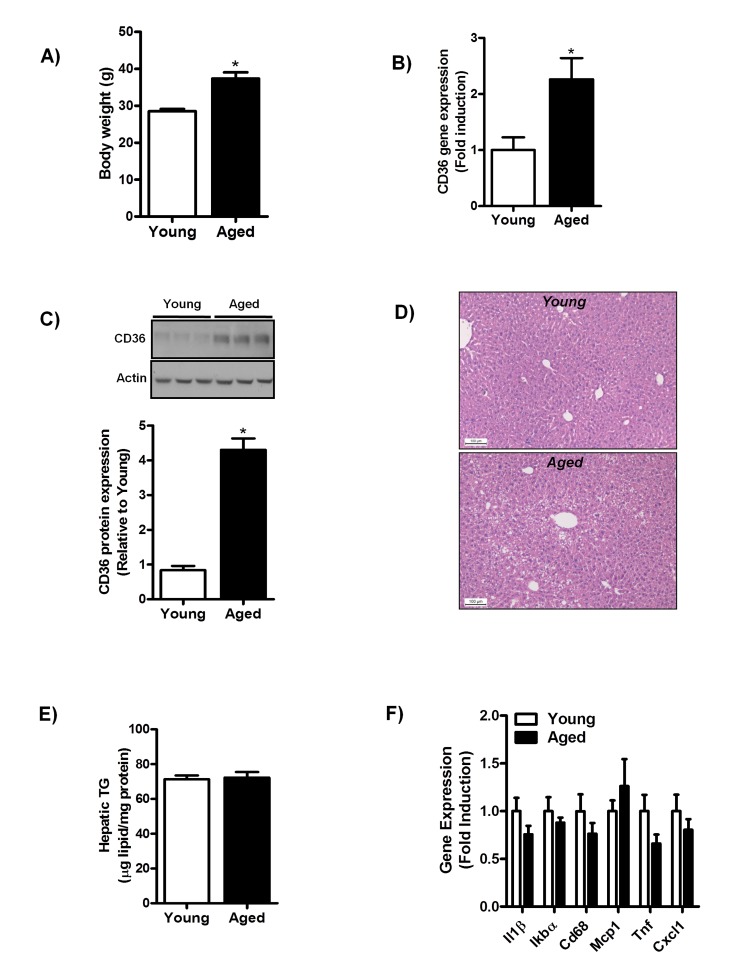
Aging does not trigger the development of hepatic steatosis and inflammation in mice fed a chow diet for one year (**A**) Body weight of young and middle-aged mice fed a chow diet. (**B**) qRT-PCR measurement of *CD36* transcript levels in livers of young and middle-aged mice (versus young mice) fed a chow diet, expressed as fold induction. (**C**) Immunoblot analysis using anti-CD36 and anti-actin (protein loading control) antibodies was performed on liver extracts from young and middle-aged mice fed a chow-diet. Immunoblots were quantified by densitometry and normalized against actin as a control for protein loading. (**D**) H&E staining of paraffin embedded liver sections obtained from young and middle-aged mice fed a chow diet. (**E**) Triglyceride (TG) levels were determined in livers of young and middle-aged chow-fed mice. (**F**) Hepatic gene expression of interleukin-1 (*Il1)*,IkappaB alpha (*Ikbα*), Cluster of Differentiation 68 (*Cd68*), monocyte chemoattractant protein-1 (*Mcp-1*), tumor necrosis factors (*Tnf*) and Chemokine (C-X-C motif) ligand 1 (*Cxcl1*) in livers from young and middle-aged mice (versus livers of young chow-fed mice) were determined by qRT-PCR and expressed as fold induction. Values are expressed as mean ± SEM; n = 6-7 mice in each group. *p ≤ 0.05 (nonparametric Mann-Whitney *U* test).

To further investigate the correlation between aging and hepatic CD36 expression, we assessed CD36 expression in middle-aged mice fed a chow-diet for one year. Body weight was significantly increased (Fig. 2A) in middle-aged mice and no difference was observed in the liver to body weight ratio (data not shown). Hepatic CD36 gene (Fig. 2B) and protein expression (Fig. 2C) were significantly increased with aging in mice similar to that found in normal liver biopsies of aging humans.

Histological assessment of H&E stained liver sections of young and middle-aged mice showed a slight occurrence of macrovesicular steatosis in livers of middle-aged mice (Fig. 2D). However, hepatic triglycerides (TG) levels (Fig. 2E) between young and middle-aged mice did not differ, confirming that lipid droplet formation in middle-aged mice was not quantitatively relevant. We observed no alterations in the expression of several pro-inflammatory cytokines and genes encoding for proteins involved in macrophage infiltration (*Il1β, Tnf, IkBα, Cd68, Mcp1, Cxcl1*) in middle-aged mice as compared to young mice (Fig. 2F). In addition, no difference was observed in the inflammatory signaling often associated with NAFLD in livers of middle-aged mice, as the phosphorylation status of the extracellular signal-regulated kinase 1/2 (Erk 1/2*)* was not affected by aging in these mice (Sup. Fig. 1A). These data suggest that aging is not associated with overt hepatic steatosis and/or hepatic inflammation in chow-fed mice.

### Increased susceptibility to the development of HFD-induced NAFLD with age

**Figure 3:**
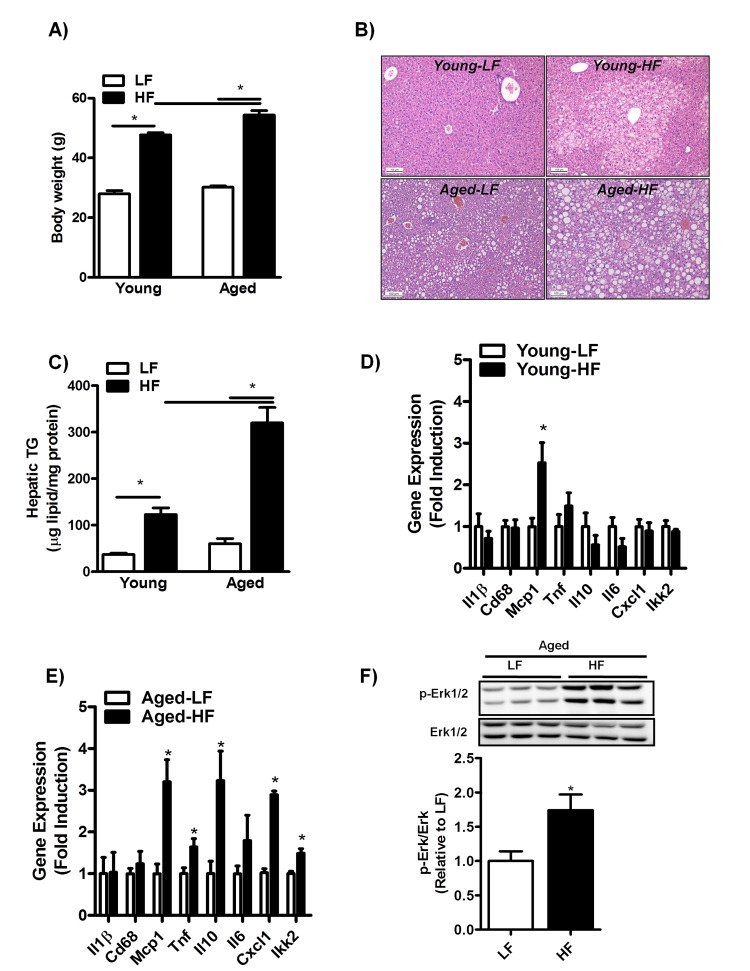
Aging increases hepatic steatosis and inflammation in mice fed a HFD for 12 weeks (**A**) Body weight of young and middle-aged mice fed a HFD. (**B**) H&E staining of paraffin embedded liver sections and (**C**) biochemically quantification of liver triglycerides (TG) of young and middle-aged mice fed a HFD. (**D**) mRNA expression of cytokines and genes involved in inflammation interleukin-1 (*Il1)*, Cluster of Differentiation 68 (*Cd68*), monocyte chemoattractant protein-1 (*Mcp-1*), tumor necrosis factors (*Tnf*), interleukin-10 (*Il10*), interleukin-6 (*Il6*), Chemokine (C-X-C motif) ligand 1 (*Cxcl1*), IkappaB kinase 2 (*Ikk2*) in the liver of young mice fed a HFD (versus LFD-fed mice) for 12 weeks determined by qRT-PCR and expressed as fold induction. (**E**) mRNA expression of cytokines and genes involved in inflammation *Il1,Cd68*, *Mcp-1*, *Tnf*, *Il10*, *Il6*, *Cxcl1*, *Ikk2* in the liver of middle-aged mice fed a HFD. (**F**) Immunoblot analysis using anti-Phospho-*Erk1/2* and anti-*Erk1/2* antibody was performed in liver extracts of middle-aged mice fed a LFD or HFD for 12 weeks, tubulin antibody was performed as a control for protein loading (not shown). Values are expressed as mean ±SEM; n = 6-8 mice in each group. *p ≤0.05 (nonparametric Mann-Whitney *U* test).

To investigate whether aging may increase susceptibility to the development of HFD-induced NAFLD, 3- and 8-month-old mice were fed a HFD for 12 weeks. While body weight was significantly increased in both young and middle-aged mice fed a HFD compared to mice fed a LFD (Fig. 3A), body weight was only marginally affected by aging in itself (Fig. 3A, HF young: 47.7 ± 0.7; HF aged: 54.4, p≤0.05). Moreover, liver to body weight ratio was significantly increased in middle-aged mice as compared to young mice fed a HFD (HF young: 2.1 ± 0.2; HF aged: 4.1 ± 0.2, p≤0.05). H&E staining of liver sections of mice fed a HFD showed the predominant presence of microvesicular steatosis in HF-fed young mice in contrast to both macro- and microvesicular steatosis in HF-fed middle-aged mice (Fig. 3B). Consistent with enhanced lipid droplet formation in middle-aged versus young mice fed a HFD, hepatic TG content was significantly affected by the superimposed effect of aging and HFD-feeding, as shown by a three-fold increase in hepatic TG levels in middle-aged compared to young mice fed a HFD (Fig. 3C). In addition, despite an increase in *Mcp1* expression levels, HFD-feeding did not elevate inflammation in the livers of young mice (Fig. 3D and Sup. Fig.1B). However, HFD-feeding was associated with a significant increase in the mRNA expression levels of *Tnf, Il10, Cxcl1 and Ikk2* (Fig. 3E) and in the phosphorylation status of Erk1/2 (Fig. 3F) in middle-aged mice. This pro-inflammatory phenotype was observed only in the livers of middle-aged mice.

### Aging alters the balance between lipid uptake and utilization in mice fed a HFD

**Figure 4:**
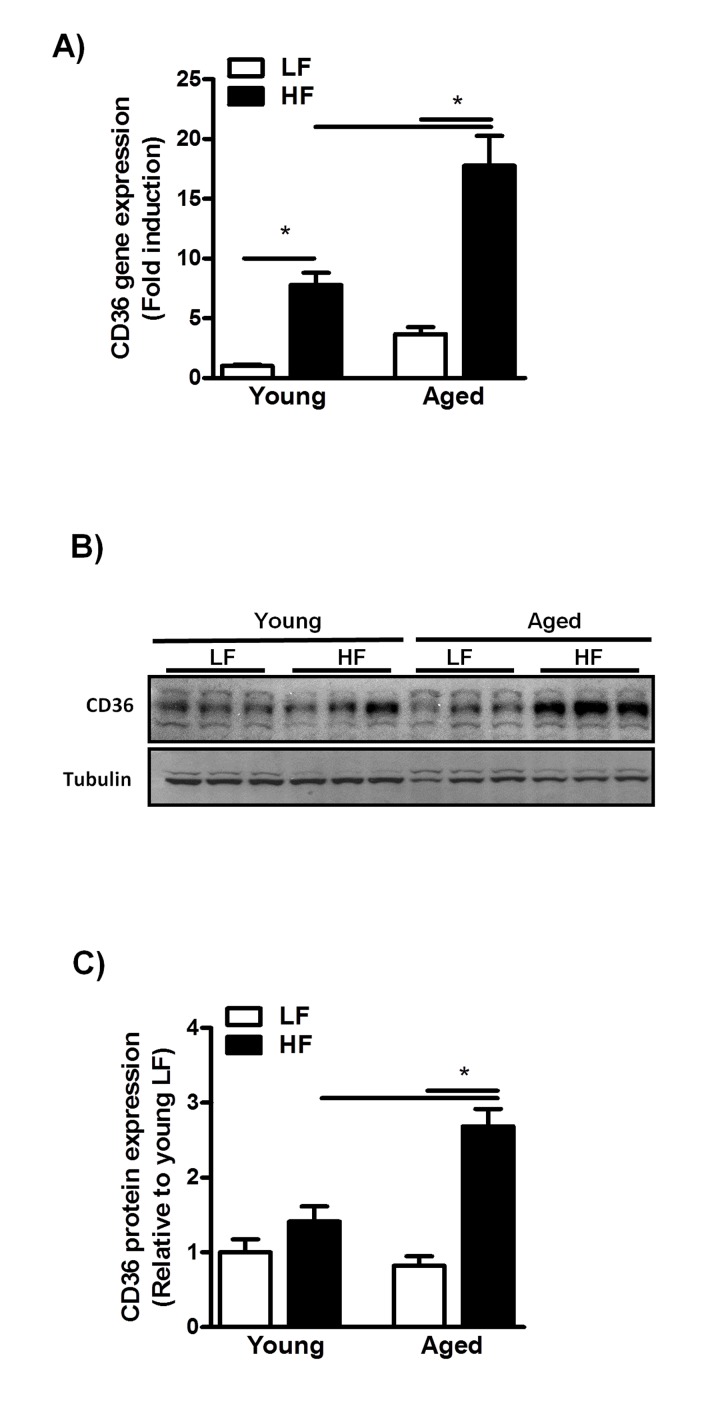
Increased CD36 expression in aged mice fed a HFD for 12 weeks (**A**) qRT-PCR measurement of Cd36 transcript levels in livers of young and middle-aged mice fed a HFD (versus young mice on LFD) and expressed as fold induction. (**B**) Immunoblot analysis using anti-CD36 and anti-tubulin antibodies was performed on liver extracts from young and middle-aged mice fed a HFD. (**C**) Immunoblots were quantified by densitometry and normalized against tubulin as a control for protein loading. Values are expressed as mean ± SEM; n = 6-8 mice in each group. * *p* ≤0.05. (nonparametric Mann-Whitney *U* test).

To identify mechanisms that may underlie this increased susceptibility to HFD-induced NAFLD, we first assessed CD36 expression levels in young and middle-aged mice fed a HFD for 12 weeks. CD36 expression was significantly increased in these mice as compared to their LFD-fed counterparts (Fig. 4A).

Moreover, CD36 gene- and protein expression levels were significantly increased by the combined effects of age and HFD feeding (Fig. 4A-C), suggesting enhanced CD36-mediated hepatic fat uptake in middle-aged mice fed a HFD. As aging has also been shown to affect mitochondrial β-oxidation through alterations in the mitochondrial electron transport chain [25–27], we next assessed the protein level of the OXPHOS complexes in both young- and middle-aged mice fed a HFD. Although feeding mice a HFD did not alter the expression of OXPHOS complexes in young and middle-aged mice, a reduction was observed in the complexes I-IV in middle-aged compared to young mice (Sup. Fig. 2A, B) suggesting an impaired β-oxi- dation in these mice. This, in combination with enhanced CD36-mediated lipid uptake, is likely to cause a severe disruption in the balance between fatty acid uptake and utilization in the livers of middle*-*aged mice, which may contribute to increased hepatic TG deposition.

### Aging increases CD36 translocation in livers from mice fed a HFD for 12 weeks

**Figure 5:**
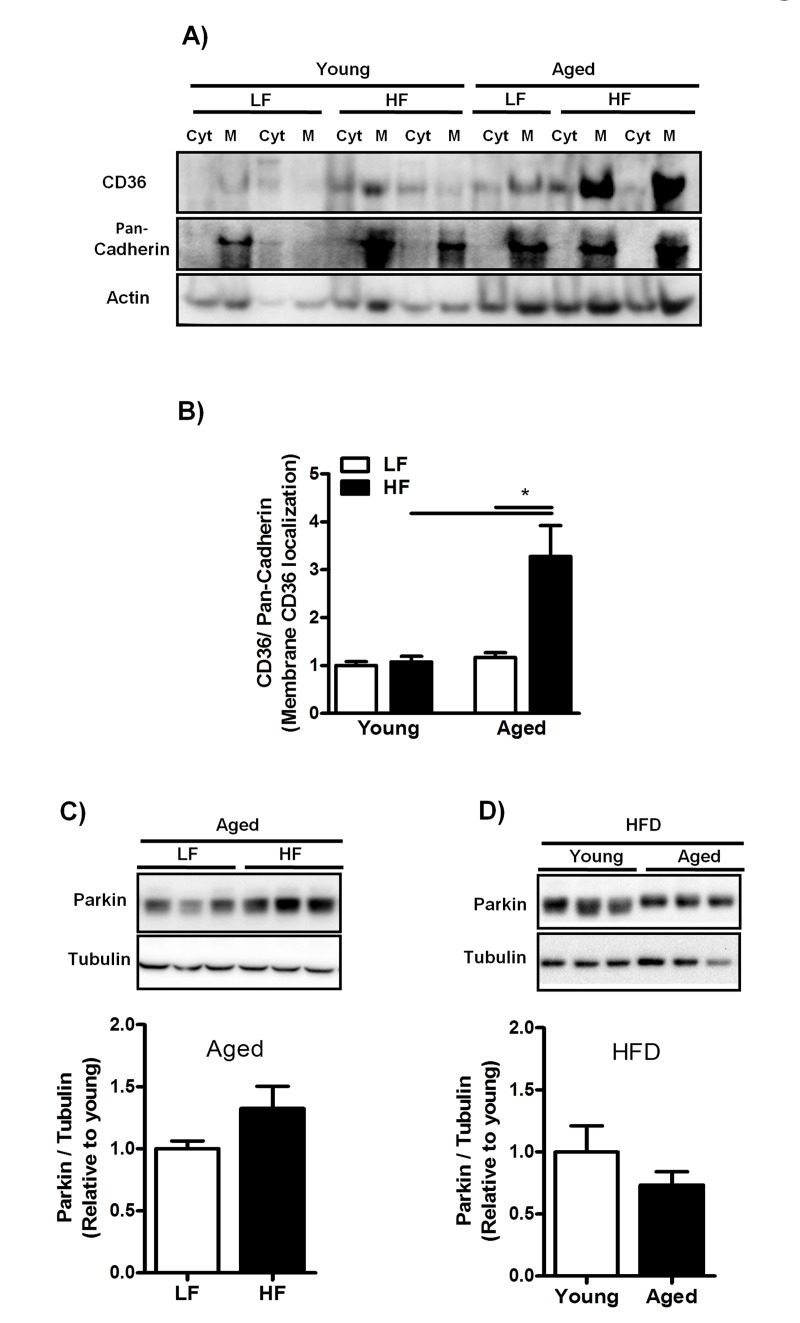
Increased plasma membrane abundance of CD36 in aged mice fed a HFD is not related to Parkin expression levels (**A**) Representative blot and (**B**) Immunoblot analysis to isolate plasma membrane (M) from cytoplasm (Cyt) using anti-CD36 antibody was performed in liver lysates from young and middle-aged mice fed a LFD and HFD. Anti-pan-Cadherin antibody was performed as a control for isolation purity and Actin antibody was performed as a control for protein loading. (**C**) Representative blot and immunoblot analysis using anti-Parkin antibody was performed in liver lysates from middle-aged mice fed a LFD and HFD and (**D**) in young and middle-aged mice fed a HFD. Tubulin antibody was performed as a control for protein loading. Values are expressed as mean ± SEM; * * *p* ≤0.05. (nonparametric Mann-Whitney *U* test).

Since enhanced efficiency of FA uptake is directly related to increased recruitment of CD36 from intracellular storage sites toward the plasma membrane [15], we next isolated membrane (M) and cytoplasmic (Cyt) fractions from livers of young- and middle-aged mice fed a LFD and HFD and measured the plasma membrane abundance of CD36 using immunoblot analysis. CD36 expression was dramatically increased in the plasma membranes of middle-aged mice fed a HFD (Fig. 5A, B) suggesting enhanced CD36 translocation in middle-aged mice on HFD. Since insulin has previously been demonstrated to induce CD36 translocation [28,29], we measured fasted insulin levels in young- and middle-aged mice fed a HFD. Plasma insulin levels were significantly increased in both young (LF: 0.077±0.19; HF: 0.66±0.09, P≤0.05) and middle-aged mice (LF: 0.18±0.02; HF: 1.34±0.15, P≤0.05) fed a HFD compared to mice fed a LFD. However, insulin levels were significantly increased in middle-aged mice compared to young mice fed a HFD. Parkin expression was assessed in both young and middle-aged mice fed a HFD as it has been reported to stabilize CD36 at the plasma membrane [30]. Parkin expression, however, was not altered in middle-aged mice fed a HFD compared to both groups: middle-aged mice fed a LFD (Fig. 5C) and young mice fed a HFD (Fig. 5D).

## DISCUSSION

In this study, we explored the role of CD36 in the development of age-associated NAFLD. Consistent with pre-clinical data [18,20,21], we report for the first time that CD36 expression is positively associated with age in biopsies from patients with normal livers. In addition, CD36 expression was elevated in biopsies from patients with non-alcoholic steatosis (NAS). However, this increase in CD36 protein expression was not further elevated in aged NAS patients. Instead, aged NAS patients displayed enhanced plasma membrane expression of CD36 as compared to young NAS patients. This is suggesting that in elderly individuals with NAS, enhanced CD36 translocation may contribute to hepatic steatosis and NAFLD as opposed to absolute CD36 protein levels. In agreement with this, middle-aged mice exhibit an increased susceptibility to the development of HFD-induced NAFLD, which is mediated by an increased abundance of CD36 on the plasma membrane. Thus, our data indicate that enhanced CD36-mediated hepatic fat uptake may contribute to an accelerated progression of age-related NAFLD in both mice and humans.

Our data are in line with previous findings confirming a role for CD36 in hepatic fatty acid uptake and hepatic steatosis in both rodents [18,31,32] and NAFLD patients [24]. Our data also extend previous findings on the pathogenic role of CD36 in the metabolic syndrome and age-related cardiomyopathy and lipotoxicity in heart and skeletal muscle [19–21] and suggest that CD36 is also one of the key candidate proteins playing a role in the development of age-related NAFLD. However, the mechanism(s) behind increased expression of CD36 in human and rodent livers is not known. Aging is associated with an increase in body weight and a physiological increase in lipid accumulation in non-adipose tissues [6]. In addition, aging increases the circulating levels of insulin, glucose and fatty acids in the human population and in mice [33], factors previously reported to either increase cellular CD36 expression or induce CD36 translocation to the plasma membrane [34–36]. As the patients in each age subgroup were matched in terms of sex distribution, body mass index, insulin resistance score (HOMA) and histological grade of steatosis (in the case of the NAS group), these parameters do not explain the differences seen in CD36 expression in our human study. Furthermore, not one single factor is likely to explain the age-related increase in hepatic CD36 levels in the normal liver group as we observed a significant increase in waist perimeter, glucose, AST and ALP levels between young and aged normal liver individuals (Table 1). In addition, we did not observe an age-effect on the hepatic CD36 expression level in NAS liver biopsy samples (Fig. 1). This is most likely due to the fact that CD36 expression levels were already significantly increased in biopsies from young NAS livers as compared with those from young normal livers. However, in young NAS patients, CD36 immunoreactivity was restricted mainly to the cytoplasm of hepatocytes, whereas in aged NAS patients, CD36 staining was largely detected at the plasma membrane of liver cells (Fig. 1, lower panels, inserts). Nevertheless, we cannot exclude the possibility that this membraneous staining pattern of CD36 might be related to accumulation of fat in the cytoplasm that may have pushed CD36 to the side; the factors responsible for the increased membrane abundance of CD36 in aged NAS patients remain elusive and further studies are required. Of all parameters tested only ALP was significantly increased in aged versus young NAS livers, but its level was within the normal clinical range. Further studies are clearly needed to explain the increased translocation of CD36 in aged NAS patients.

Increased hepatic CD36 membrane expression was predominantly observed in middle-aged mice fed a HFD. The mechanism by which CD36 is translocated to the plasma membrane has not yet been elucidated. However, it has been demonstrated that CD36 can be translocated rapidly from intracellular sites to the plasma membrane in response to insulin and thereby enhance FA uptake [28]. Therefore, the enhanced CD36 membrane expression in HF-fed middle-aged mice may be related to significantly enhanced plasma insulin levels in middle-aged compared to young mice fed a HFD. Indeed, insulin levels were elevated 7.5-fold in middle-aged mice fed a HFD in comparison to a 1.5-fold increase in the young mice. This may have contributed to a strong lipogenic drive with age, explaining not only the increased membrane abundance of CD36 but also the increased hepatic steatosis following HF-feeding in middle-aged mice (Fig. 3B and C). In addition, increased plasma insulin levels have also been observed in senescence-prone (SAMP10) mice and reported to explain the increased lipid accumulation with age in these mice [11]. However, since cytokines have been demonstrated to regulate CD36 expression at the transcriptional level [37], the possibility also arises that increased cytokine levels during hepatic inflammation (Fig. 3F) are responsible for the enhanced CD36 membrane abundance observed in middle-aged mice fed a HFD (Fig. 5A, B). Consistent with this is the hepatic overexpression of CD36 in NASH patients and in those with chronic HCV G1 infection [24]. Nevertheless, we cannot exclude the role of other mechanisms such as reduced CD36 recycling resulting in a delay in the proteasomal degradation of CD36 [38]. Indeed, CD36 turnover has been shown to be abnormally slow in macrophages from insulin-resistant ob/ob mice, resulting in CD36 accumulation at the plasma membrane [39]. In line with this, insulin has been shown to reduce CD36 ubiquitination and enhance CD36 protein levels in CHO cells expressing both the insulin receptor and CD36 [38]. As this was associated with enhanced fatty acid uptake, it may provide an alternative mechanism for trapping CD36 at the membrane surface. Furthermore, Parkin is suggested to play a pivotal role in enhancing hepatic fat uptake by increasing the protein stability and half-life of CD36 through monoubiquitination of CD36 [30]. However, we did not observe an age- or HFD-associated increase in Parkin expression in middle-aged mice compared to young mice. Nevertheless, more recent work has shown that neddylation of Parkin increases E3 ligase activity without affecting expression [40]. This may be an alternative pathway leading to increased mono-ubiquitination of CD36 and stabilization at the plasma membrane and warrants further study. Altogether, the exact mechanism by which CD36 is translocated to the plasma membrane has not been fully elucidated and needs further investigation.

In addition to our data implicating CD36 in age-induced NAFLD, we also report that middle-aged mice show a decline in the protein expression levels of the mitochondrial electron transport chain protein expression (complex I-IV), suggesting that fat oxidation is impaired in middle-aged mice irrespective of HF-feeding. Although we did not perform direct measurements of fatty acid oxidation, this is in line with a previous study showing that mitochondria of middle-aged mice display many electron transport chain defects and decreased complex I and IV activities [41]. Our results may thus suggest that the CD36-mediated increase in hepatic fat uptake in HFD-fed middle-aged mice, coupled with a decrease in fat oxidation in the liver, will enhance triglyceride synthesis in the liver during aging and thus may be key to advancing NAFLD with aging.

Furthermore, our results indicate that minor lipid droplet formation is indeed present in middle-aged mice fed a regular chow diet for one year (Fig. 2D). However, the livers of these mice did not accumulate more TG (Fig. 2E) and failed to show hepatic inflammation (Fig. 2F, Sup. Fig. 1A), indicating the absence of NAFLD. Moreover, NAFLD development (hepatic steatosis and inflammation) occurred only in middle-aged mice fed a HFD (Fig. 3). Our data thus suggest that the aging process in itself does not accelerate NAFLD development in the rodent liver; however, aging does greatly promote the development of hepatic steatosis and inflammation (NAFLD) when superimposed with a HFD. Since hyperinsulinemia, steatosis and inflammation are considered as systemic manifestations of cellular hyperfunction or overactivity [42,43], our findings are consistent with the hyperfunction theory of aging proposed by Blagosklonny, which postulates that processes contributing to growth and reproduction run on in later life, leading to pathology and eventually to death [42,43].

Although our findings seem to contradict the current understanding that aging is associated with hepatic steatosis in humans and rodents, they are in line with the recent study of Fontana [14], who showed that aged mice are more prone to develop diet-induced steato-hepatitis than young mice. Significantly, the authors did not find a difference in the development of hepatic steatosis between 6-, 12- and 22-month-old mice fed a HFD for 16 weeks [14]. Although the authors did not study the effect of aging on hepatic lipid accumulation in chow fed mice, this clearly contrasts with our observation that HF-feeding when super-imposed on middle-aged mice does significantly promote hepatic steatosis. The reason for this discrepancy is not clear; it may, however, be related to the extended length of the dietary period in the study of Fontana *et al*. [14]. This could explain the lower accumulation of hepatic lipid levels in the young mice fed a HFD in our study.

In summary, we have shown that aging *per se*does not contribute to the development of NAFLD; however, it does increase susceptibility to the development of diet-induced NAFLD. Furthermore, we report for the first time that aging increases CD36 membrane expression in the livers of both mice and humans. Therefore we propose that increased CD36 localization/stabilization at the plasma membrane may be a key to enhanced hepatic fat uptake, and may play an important role in advancing NAFLD with age. Our results suggest that drugs that prevent the increase in CD36 expression and/or CD36 from anchoring at the plasma membrane may alleviate NAFLD and prevent the transition from benign steatosis toward more advanced NASH.

## EXPERIMENTAL PROCEDURES

### Patients

This study comprised 56 non-diabetic patients who underwent a needle liver biopsy during programmed laparoscopic cholecystectomy. Liver biopsy sections were collected as previously described [24] and histological diagnosis of NAFLD according to Brunt *et al* [44] was used to select patients with histologically normal livers (grade 0, < 5% of steatotic hepatocytes; n=30) or with simple steatosis (NAS, grades 1 and 2, 5 to 66% of steatotic hepatocytes; n=26). Each group was further classified according to distinct age categories, resulting in a young subgroup aged 20-42 years (normal liver: n=16, NAS: n=11) and an older subgroup aged 50-83 years (normal liver: n=14; NAS: n=15). Patients in each age subgroup were matched in terms of sex distribution, BMI, insulin resistance score (HOMA) and histological grade of steatosis (in the case of NAS group). Since hepatic inflammation is a confounding variable [24], NASH patients were excluded from the study. Additional standard exclusion criteria were: patients with an alcohol intake of more than 20 gram/day, those with chronic hepatitis B, C or HIV infections, and patients taking chronic medications. The study was performed in agreement with the Declaration of Helsinki and with local and national laws. The Human Ethics Committee of the University Hospital Santa Cristina (Madrid, Spain) approved the study procedures, and each patient provided written informed consent before inclusion in the study.

### Immunohistochemistry on human liver biopsy sections

Immunostaining and computational image analysis using analySIS® software (Soft Imaging System, Gmbh, Münster, Germany) were performed on formalin-fixed paraffin-embedded liver biopsy sections (4 m thickness) from all patients of each group, as previously described [24]. All immunostained liver biopsy sections were first coded and then evaluated by an expert in liver immunohistochemistry (CG-M). By measuring the stained surface in 6 different lobular areas, an average score representing the percentage of liver tissue area occupied by CD36 staining was obtained. In order to more accurately evaluate the expression of CD36 restricted to hepatocytes, we measured CD36 staining by using a high magnification objective (40x) (Nikon, Tokyo, Japan) to focus on lobular areas where hepatocytes are the predominant cell type. For each liver biopsy, the average value was considered as the hepatic CD36 expression index and expressed as the percentage of lobular area occupied by CD36 staining.

### Clinical and laboratory assessment

After a 12 h overnight fast, clinical and anthropometric data as well as venous blood samples were obtained from each patient at the time of liver biopsy. All serum chemistry analyses were performed at the Central Laboratory Service of the University Hospital Santa Cristina (Madrid, Spain) as described [24]. Insulin resistance was calculated by the homeostasis model assessment (HOMA-IR) [45]. Antibodies against (hepatitis B, C and HIV) surface antigens were tested for by immunoenzymatic assays (Murex, Dartford, UK) and were used to exclude patients from the study.

### Mice

This study was performed with the approval of the University of Alberta Animal Policy and Welfare Committee and the University of Groningen Ethics Committee for Animal Experiments, which adheres to the principles and guidelines established by the European Convention for the Protection of Laboratory Animals. Experiments were carried out on young (12-14 week old) and middle-aged (52-58 week old) male C57BL6/J mice, maintained on a 12-hour light-dark cycle and given free access to water and chow diet unless otherwise stated. At 12-14 or 32-34 weeks of age, a subset of mice was randomly divided into a low-fat diet (LFD) group (D12450B, 10% kcal from fat, Research Diets) and a high-fat diet (HFD) group (D12492, 60% kcal from fat, Research Diets) for a period of 12 weeks. Insulin was measured in plasma from fasted mice as described [20].

### Liver histology

Paraffin-embedded sections of the liver (4μm) were stained with hematoxylin-eosin (H&E). Microscopy was performed with a Leica DM 3000 microscope with a DFC420 camera (Leica Microsystems, Rijswijk, the Netherlands).

### Quantitative real-time(qRT) PCR— Hepatic gene expression analyses

Total RNA was isolated from the liver using QIAazol reagent (QIAGEN, Venlo, the Netherlands). First-strand cDNA synthesis was performed using the QuantiTect Reverse Transcription Kit (QIAGEN, Venlo, the Netherlands). qRT-PCR was performed using a 7900HT System (Applied Biosystems, Warrington, UK) and values were corrected using the housekeeping gene Cyclophillin A (*Ppia*). Primers are presented in Supplemental Table 1.

### Determination of liver triglycerides

Crushed liver tissues from fasted mice were homogenized, lipids extracted and the amount of triglyceride determined by gas-liquid chromatography as previously described [46].

### Immunoblot analysis

Liver tissue was homogenized and equal amounts of protein were subjected to SDS-PAGE, transferred to nitrocellulose or PVDF membrane (Amersham, Buckinghamshire, UK) and immunoblotted with antibodies against, Erk1/2, p-Erk1/2, Actin, α-tubulin, Parkin (Cell Signaling Technology, Leiden, the Netherlands), Pan Cadherin (Abcam, Cambridge, UK), CD36 (Novus Biologicals, Littleton, USA) and Oxphos (Abcam, Cambridge, UK; a kind gift from Dr. Herman Sillje, UMCG, Groningen). Immuno-positive bands were visualized by chemiluminescence (GE Healthcare Life Sciences, Diegem, Belgium) and quantified using the Molecular Imager ChemiDoc xrs+system from Bio-Rad (Veenendaal, the Netherlands).

### Statistical analysis

Data are presented as mean ± SEM unless stated otherwise. The characteristics of the patients studied were compared by the Pearson *2* test or Fisher exact test for categorical variables and the unpaired *t* test or Mann-Whitney *U* test for continuous variables. Spearman's *rho* test was used to evaluate correlations. Experimental data were compared using the unpaired *t* test or Mann-Whitney *U* test. The level of significance was set at P<0.05. All statistical analyses were performed in GraphPad Prism (v. 5.00 for Windows, San Diego, CA).

## SUPPLEMENTAL FIGURES AND TABLES



## Abbreviations

(NAFLD): Nonalcoholic fatty liver disease
(NAS): nonalcoholic steatosis
(NASH): nonalcoholic steatohepatitis
(HFD): high-fat diet
(LFD): low-fat diet
(qRT-PCR): Quantitative real-time polymerase chain reaction
(TG): triglycerides
(OXPHOS): oxidative phosphorylation
(HCV G1): hepatitis C virus infected with genotype 1
