# Supplementary material for: Increased hepatic CD36 expression with age is associated with enhanced susceptibility to nonalcoholic fatty liver disease
Source: Aging (Albany NY). 2014 Apr 9;6(4):281–95. doi: 10.18632/aging.100652 (PMC4032795; doi:10.18632/aging.100652)
Supplement: Supplementary file 1 [file aging-06-281-s001.pdf]

grading and staging the histological lesions. Am. J. Gastroenterol. 1999;94:2467–2474.
45. Matthews DR, Hosker JP, Rudenski AS, Naylor BA, Treacher DF, Turner RC. Homeostasis model assessment: insulin resistance and beta-cell function from fasting plasma glucose and insulin concentrations in man. Diabetologia. 1985;28:412–419.
46. Jacobs RL, Devlin C, Tabas I, Vance DE. Targeted deletion of hepatic CTP:phosphocholine cytidylyltransferase alpha in mice decreases plasma high density and very low density lipoproteins. J. Biol. Chem. 2004;279:47402–47410.

SUPPLEMENTARY DATA

Supplementary Table 1. Primer sequences used for qRT-PCR

Gene	Forward primer 5'-3'	Reverse primer 5'-3'
Ikk2	GGAGTACTGCCAAGGAGGAGAT	ACAGGCTGCCAGTTAGGGAGGAAG
IkBα	TGGAAGTCATTGGTCAGGTGAA	CAGAAGTGCCTCAGCAATTCCT
Cd68	TGACCTGCTCTCTCTAAGGCTACA	TCACGGTTGCAAGAGAAACATG
CxcL1	CCAAACCGAAGTCATAGCCAC	GTCTTCTTTCTCCGTTACTTGG
Il10	GCTCTTACTGACTGGCATGAG	CGCAGCTCTAGGAGCATGTG
Il-1β	TGCAGCTGGAGAGTGTGG	TGCTTGTGAGGTGCTGATG
Il-6	GTTCTCTGGGAAATCGTGGA	TTTCTGCAAGTGCATCATCG
Mcp1	GCTGGAGAGCTACAAGAGGATCA	ACAGACCTCTCTCTTGAGCTTGGT
Cd36	GATCGGAACTGTGGGCTCAT	GGTTCCTTCTTCAAGGACAACTTC
Tnf	GTAGCCCACGTCGTAGCAAAC	AGTTGGTTGTCTTTGAGATCCATG
Ppia	TTCCTCCTTTCACAGAATTATTCCA	CCGCCAGTGCCATTATGG

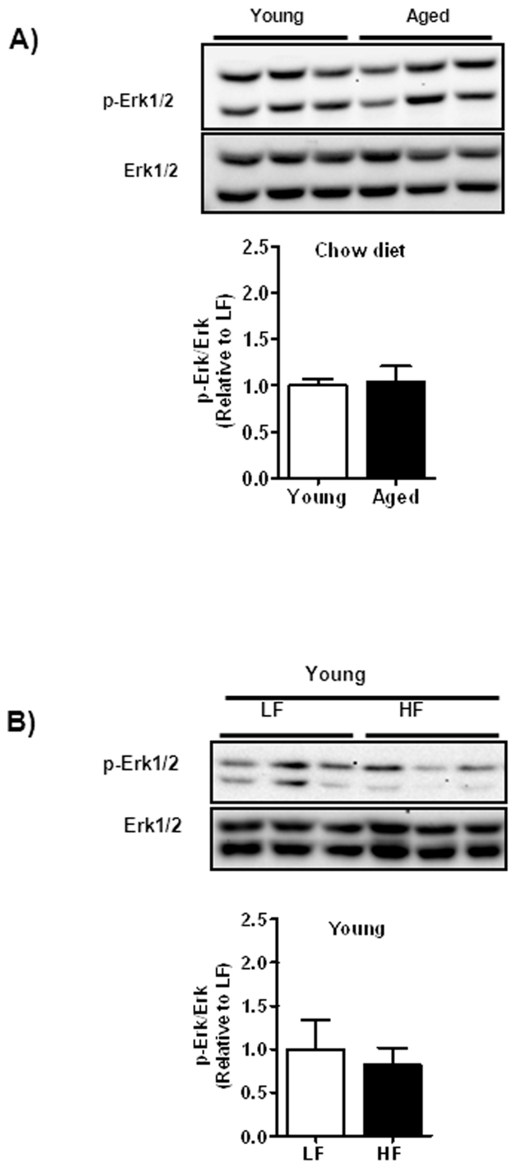

Supplementary Figure 1. Hepatic inflammation did not altered in the aged mice fed a chow diet and young mice fed with HFD for 12 weeks. (**A**) Phosphorylation status of Erk1/2 was detected in liver extracts from young and middle-aged mice using immunoblot analysis. (**B**) Immunoblot analysis using anti-phospho-Erk1/2 and anti-Erk1/2 antibody was performed in liver extracts from young mice fed a HFD for 12 weeks. Values are expressed as mean ± SEM; n = 6–8 mice/group. *p ≤0.05 (Nonparametric Mann-Whitney U test).

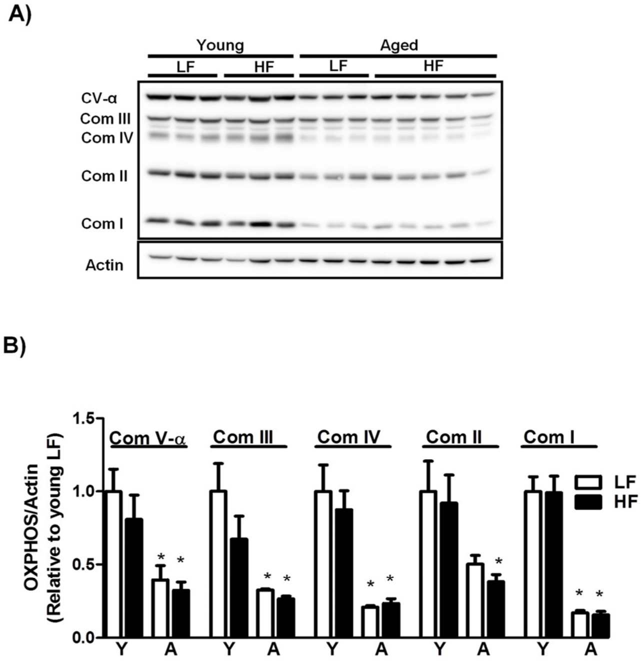

Supplementary Figure 2. Reduced expression of oxidative phosphorylation protein complexes (OXPHOS) in middle-aged mice. (**A**) Representative immunoblots from liver extracts of young (Y) and middle-aged (A) mice fed a LFD and a HFD using anti-OXPHOS and actin antibody (**B**) Immunoblots were quantified by densitometry and normalized against actin as a control for loading. Values are expressed as mean ± SEM; n= 6-7 mice/group. *p≤ 0.05. (Nonparametric Mann-Whitney U test).
